# Effect of bolus enteral tube feeding on body weight in ambulatory adults with obesity and type 2 diabetes: a feasibility pilot randomized trial

**DOI:** 10.1038/s41387-020-0125-6

**Published:** 2020-06-17

**Authors:** E. O. Beale, W. Lee, A. Lee, C. Lee, E. Soffer, P. F. Crookes, K. Eagilen, R. Chen, W. J. Mack, H. Tong

**Affiliations:** 1 Division of Endocrinology and Diabetes, Keck School of Medicine, University of Southern California, Los Angeles, 90033 CA USA; 2 Division of Gastroenterology, Keck School of Medicine, University of Southern California, Los Angeles, 90033 CA USA; 3 Division of Foregut Surgery, Keck School of Medicine, University of Southern California, Los Angeles, 90033 CA USA; 4 Edward R. Roybal Comprehensive Health Center Dental Clinic, East Los Angeles, 90022 CA USA; 5 Department of Preventive Medicine, Keck School of Medicine, University of Southern California, Los Angeles, 90033 CA USA; 6 Herman Ostrow School of Dentistry University of Southern California, Los Angeles, 90033 CA USA

**Keywords:** Type 2 diabetes, Obesity, Translational research, Randomized controlled trials, Weight management

## Abstract

**Background/objectives:**

To ascertain the effect on body weight of 14 days of bolus enteral feeding with mixed meal (MM) and electrolyte solution (ES) in ambulatory adults with type 2 diabetes and obesity, and also the safety and feasibility of using a modified, intraorally anchored enteral feeding tube for this purpose.

**Subjects/methods:**

We conducted a randomized, crossover pilot trial with 16 participants. A 140 cm, 8-French feeding tube was placed in the jejunum under electromagnetic guidance and anchored intraorally. Participants were randomized to self-administer 120 mL 523 kJ (125 kcal) MM, or 50 kJ (12 kcal) ES four times/day for 14 days. After ≥14 days without the tube, participants crossed over to the other treatment. The primary outcome compared weight change between treatments. Thereafter, participants could elect to undergo additional MM cycles. Participants were encouraged to continue with all usual activities including eating ad lib throughout the study.

**Results:**

Ten participants withdrew prior to completing two randomized 14-day cycles (4 social, 3 intolerant of anchor, and 3 intolerant of tube). Six participants were assessed for the primary outcome and showed no significant difference in weight loss between MM and ES (*p* = 0.082). For the secondary outcome of within-group weight loss, average weight loss from baseline was significant for MM but not for ES: −2.40 kg (95% CI: −3.78, −1.02; *p* = 0.008) vs. −0.64 kg (95% CI: −2.01, 0.74; *p* = 0.27). A total of 23 2-week cycles were completed (12 paired, 2 unpaired, and 9 additional), with no significant adverse events for 334 days of tube use.

**Conclusions:**

Repeated bolus nutrient administration via enteral feeding tube is associated with weight loss in adults with obesity and type 2 diabetes, with no significant difference seen between MM and ES feeds. The prototype device was safe, but requires development for further investigation into the effect of bolus jejunal feeding on weight and to improve acceptability.

## Introduction

Gastric bypass surgery (GB) is one of the most effective therapies available for obesity and related comorbidities, including type 2 diabetes^1–3^. GB and other metabolic weight-loss procedures, however, are unavailable to most individuals who could benefit from this intervention, largely due to cost and safety concerns^4,5^. Non-surgical methods that simulate the weight-loss promoting mechanisms of GB could allow for wider availability of its benefits^6,7^.

It is now generally accepted that the benefit of GB is largely due to the activation of multiple synergistic systemic and central pathways^8–10^. The rationale for this pilot study was to evaluate, in a minimally invasive manner, one consequence of GB, namely repeated rapid delivery of nutrient directly to the jejunum. We aimed to evaluate the effect of this intervention on body weight in adults with obesity.

This work is based on extensive prior research in patients undergoing bariatric surgery suggesting that a key initiating mechanism for weight loss following GB surgery is delivery of nutrient rapidly and directly to the jejunum, with added glycemic benefit with foregut bypass^3,8,11,12^. This increase in the post-pyloric nutrient delivery rate has been shown to stimulate the release of multiple appetite-suppressing gut hormones, change nutrient and stretch sensing, vagal activation, and alter bile metabolism in a manner that promotes a fed physiological state and reduces food intake^3,8–15^.

Several human studies have used enteral feeding tubes to identify nutrients, doses, sites, and rates of administration that are associated with alterations in appetite-suppressing gut hormones and net reduction in caloric intake^13–19^. Most have evaluated the effect of a single dose of nutrient, but some have lasted up to 3 days^20,21^. We demonstrated in adults with obesity and type 2 diabetes that the rapid administration of a single mixed-meal (MM) bolus to the upper intestine via nasoenteral tube was associated with increased circulating levels of GLP-1, PYY, insulin, and a suppression of appetite that was greater than that seen when the same MM was taken orally^22^.

We hypothesized that repeated administration of nutrient boluses directly to the jejunum would promote weight loss. The aim of this pilot study was to ascertain the effect of a 14-day bolus jejunal feeding with MM and electrolyte solution (ES) on the body weight of ambulatory adults with type 2 diabetes and obesity. In order to achieve this aim, a simple modified enteral feeding tube-based device was devised, allowing participants to continue with their usual daily activities without the tube being evident to others, while self-administering nutrient several times a day. We also aimed to assess the feasibility and safety of using the modified tube to test the hypothesis.

We present here the first-in-human findings of the feasibility and safety of the prototype device as well as our experience evaluating the hypothesis that bolus jejunal feeding promotes weight loss.

## Subjects and methods

### Trial design

**Fig. 1:**
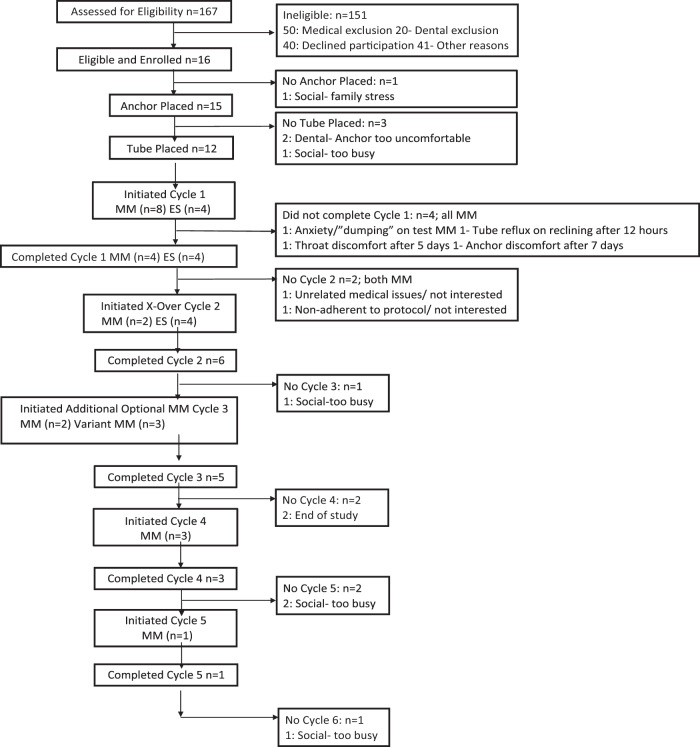
Participant flow. MM mixed-meal cycle, ES electrolyte solution cycle.

This study was initially designed as a prospective, randomized, parallel 2-group, 28-day clinical trial. However, the requirement for a healthy upper molar for secure and comfortable placement of the novel intraoral anchor significantly slowed enrollment (see Fig. 1). Therefore, Institutional Review Board approval was obtained to change the study to a randomized, crossover design. Free-living ambulatory adults with obesity and type 2 diabetes were randomized 1:1 to administer a 120 mL of bolus feed (“treatment”) via an orojejunal tube four times/day for 14 days: 120 mL of 523 kJ (125 kcal) MM or 50 kJ (12 kcal) ES. All other aspects of each cycle were the same. After 14 days, the tube was removed, and the participants were followed for 14 more days to complete the first cycle. They were then requested to repeat the 28-day cycle with the other treatment. Participants who completed two randomized 28-day cycles of MM and ES were invited to undergo further cycles of MM, or variations of MM, which provided additional data regarding the longer-term tolerance and safety of the tube. All institutional and federal regulations concerning the informed consent process and trial conduct were followed. The study was conducted in adherence to ICH Good Clinical Practice.

### Participants

Major inclusion criteria: 18–70 years of age, type 2 diabetes on diabetic medication, BMI > 30 kg/m^2^, average capillary or interstitial glucose <200 mg on 2–4 times daily self-monitored glucose readings in the week prior to enrollment and evaluated by a dentist as having acceptable dental hygiene and dentition.

Major exclusion criteria: Use of DPP-IV inhibitors or GLP-1 receptor analogs, conditions that could prevent spontaneous passage of an 8-French (Fr) jejunal tube if it were to dislodge distally, active esophagitis, known hiatal hernia, at risk for gastrointestinal bleeding, other major illness, pregnant or potentially fertile, unable to adhere to study protocol, and known eating disorders.

Setting: All study visits were outpatient visits at the LAC-DHS USC-affiliated Edward R. Roybal Comprehensive Health Center in East Los Angeles. This clinic mostly serves medically uninsured and underinsured Hispanic patients with limited access to dental and medical care, including behavioral-modification weight-loss programs, weight-loss pharmacotherapy, and metabolic surgery.

### Device

Intraoral anchor: A custom-made cylindrical stainless-steel metal channel was welded on the buccal surface of a plain, stainless steel, premade orthodontic molar band for a maxillary first molar (MR^TM^1 American Orthodontics; Sheboygan, Wisconsin, USA).

Tube: An 8 Fr 140 cm CORTRAK tube (#20–9558TRAK2; Avanos, Alpharetta GA) was modified by removing the dual-access port, leaving only an ~4-mm flange that secured the tube on the anchor. A custom-made stainless-steel cap sealed the tube when it was not in use.

Treatment administration set: Treatment was administered via a 60-cc syringe with a blunt tapered plastic orthodontic-grade syringe tip and flushed with 10-cc plain water after each use. Participants were advised that if they experienced difficulty administering treatment that this was likely due to potential kinking of the tube near the tip. They were advised to partially withdraw the tube (~15 cm) and gently re-insert it.

### Interventions

Day −14: The study dentist placed orthodontic dental separators around a select upper molar. The participant was asked to self-monitor glucose levels at least four times daily.

Day −7: A size-fit intraoral anchor was cemented to the selected upper molar.

Day 0: An orojejunal tube was placed and position confirmed following standard technique under electromagnetic guidance using the CORTRAK* 2 Electronic Access System (Avanos Medical Devices; Alpharetta GA, USA). Participants were fully alert during tube placement and no sedation, anesthesia, or X-rays were used. The participant was then randomized to MM or ES. A test treatment bolus was administered under PI supervision.

Days 0–14: Participants were requested to eat and drink ad lib throughout the study and to continue with their usual daily activities. They were asked to follow the protocol as closely as conveniently possible. MM or ES was to be given over a minimum of 5 min and a maximum of 20 min, to a maximum volume of 125 mL four times throughout the day, typically about 30 min before breakfast, lunch, late afternoon snack or meal, and evening snack or meal. In order to help maintain hydration during tube use, all participants took 1000 mL ES daily. During MM cycles, participants took the full 1000 mL orally, while during ES cycles 500 mL was taken by tube (as treatment) and 500 mL by mouth to help maintain hydration.

Days 15–28: The participant maintained a symptom and glucose log for 14 days after the removal of the tube.

Days 0, 7, 14, 21, 28: At weekly study visits, clinical review was conducted, outcome measurements taken, and study supplies given. The participant was seen in the fasted state.

### Treatments and rationale

MM: Ensure Nutrition Shake^®^ Abbott Park, Illinois, USA [Per 250 mL bottle: 920.5 kJ (220 kcal) protein 9 g, carbohydrate 33 g (15 g sugar), fat 6 g, sodium 190 mg, potassium 390 mg]. This treatment was selected as we previously demonstrated a significant increase in GLP-1, PYY, and insulin levels, along with appetite suppression, with single dose administration to the upper intestine in adults with obesity and type 2 diabetes^22^.

ES: Unflavored Pedialyte^®^ Abbott Park, Illinois, USA [osmolality, 250 mOsm/kg H_2_O; per liter: sodium, 45 mEq; potassium, 20 mEq; chloride, 35 mEq; zinc, 7.8 mg; dextrose, 25 g; 418.4 kJ (100 kcal). No artificial sweeteners or flavors]. This treatment was selected to serve as a low-calorie control.

### Glucose management

Participants were asked to self-monitor glucose four times a day, and when flash glucose monitors became available these were provided to participants. The symptoms and signs of hypoglycemia were reviewed with the participants who were also monitored at least weekly throughout the study by the PI, an experienced clinical diabetologist. Participants were requested to contact the study team at any time throughout the study with any concern, including concerns regarding their blood glucose. Intermediate and long-acting doses of insulin were reduced on initiation of the intervention by one-third to one-half, and rapid-acting insulin and sulfonylurea discontinued. Doses were reviewed and adjusted at weekly study visits at the discretion of the clinician PI. This management was based on protocols used in other diabetes studies and expected calorie restriction, and our management was similar to that recommended for marked dietary caloric restriction in individuals with type 2 diabetes^23^.

### Outcome measures

The original approved study design included multiple primary and secondary anthropometric, dietary, and biochemical outcome measures as described in the protocol. However, this proved very time-consuming, resulting in the provision of incomplete data by participants. We thus greatly reduced the requirements of the participants while continuing to monitor them closely for clinical safety. After commencement of this pilot trial, emphasis changed to focus on simpler measures of participant flow and body weight measurement, and identifying reasons for early withdrawal from the trial, in order to design an appropriately powered and definitive weight-loss trial. The primary outcome was 14-day weight change (from cycle baseline to end of the 14-day nutrient intervention: days 0–14). Secondary outcomes included the 28-day cycle weight change (cycle baseline to end of cycle: days 0–28), and the weight change from the 14-day end of nutrient intervention to 28-day end of cycle (days 14–28). For all enrolled participants, select baseline demographic and clinical parameters were recorded. Major events related to device use, and significant clinical events, including adverse events, were documented at weekly study visits. Body weight was measured in the morning with the participant wearing light clothing on a calibrated scale.

### Informed consent and blinding

Potential participants were provided with information and supplies in a manner that aimed to maximize safety and minimize risk of bias. Participants were informed that the main purpose of the study was to see if there was weight loss when people receive some of their daily nutritional requirements through a tube into the jejunum and that they might lose some weight. They were advised that they could see alterations in blood glucose level, including hypoglycemia, and that another purpose of the study was to gather information about how well people tolerated having a tube anchored in the mouth for 2 weeks. With respect to nutrients, there was no discussion as to the composition of the two treatments or hypothesized difference in response. Complete blinding of the participants by providing identical appearing tube feeds and containers was not feasible due to difficulty finding similar appearing nutrients of the desired composition and the preference to provide the feed in the original sealed container to minimize risk of contamination.

The investigators were not blinded to the treatment being administered; however, steps were taken to minimize the risk of bias. The research assistant was responsible for ascertaining randomization, preparing supplies, and assessment of the main outcome (weight), which was recorded automatically by the scale, usually with a dated printout of the value. The P.I., an endocrinologist, was responsible for participant safety, including monitoring of blood glucose levels, decisions on dosage adjustment, and withdrawal from the study. Although these duties may have been done without being aware of the assignment for this initial pilot study when the effect of long-term bolus nutrient administration was uncertain, it was considered safer for the P.I. not to be blinded to the type of study cycle.

### Sample size

In this pilot study, we anticipated a significantly greater weight loss in the MM arm with a sample size of ten participants per group, based on previous results with dietary restrictions of 5230 kJ (1250 kcal) for 1 week^24^.

Interim analysis and stopping guidelines were not initially specified. The decision was made by the investigators to close the pilot trial after sufficient data were acquired for calculating effect sizes and parameter estimates for use in the design and power calculations of future studies with an improved device prototype.

Randomization was performed using an online random number generator, without blocking or restrictions. The statistician provided the randomization list to the research coordinator who prepared sequentially numbered sealed opaque envelopes. An envelope in the numbered set was only opened by the research coordinator after confirmation of tube placement. Participants, but not the research team, were blinded to the composition of the treatment as labels were removed from the original sealed bottles being provided. The bottles and contents for both arms did not look the same, but the hypothesized effects of different treatments were not discussed with participants. Weight was measured and recorded by the research assistant. Apart from treatment, all other aspects of each cycle were the same.

### Statistical methods

For all enrolled participants, descriptive summary data, including mean (SD) for continuous variables, were summarized for select baseline demographic and clinical parameters, major events related to device use, and significant clinical events including adverse events. We used a general linear model to compare treatments for the primary outcome of 14-day weight change (days 0–14). Main effects at each cycle were specified for intervention (MM vs. ES), period (cycle 1 or 2), and randomized crossover sequence. A random term for subjects nested within sequence was also specified. The primary analysis reported the per-crossover protocol analysis of the first two randomized cycles. In the secondary analysis, the additional six MM cycles were added (a total of 18 cycles analyzed among the 6 participants). Secondary outcomes included weight change in the 28-day (days 0–28) and post 14-day (days 14–28) cycle. Paired *t* test was used to compare baseline and final measures in participants who underwent ≥2 cycles.

## Results

### Participant flow and feasibility

Recruitment for this study began in November 2014, and the study took place between November 2014 and February 2019, with the last follow-up visit in February 2019 (Fig. 1). Sixteen participants (10%) were enrolled from screening, and 12 were randomly assigned to cycle 1 treatment. Of these, six participants completed 28-day randomized cycles for both MM and ES, and were analyzed for the primary outcome. A total of 23.8 2-week cycles were completed for 334 days of tube placement. The trial ended due to the investigators’ decision to improve the device prototype and protocol rigor with insights gained from the feasibility study prior to further evaluation.

### Demographics and baseline clinical measures

Baseline demographic and clinical characteristics for all 16 participants who were randomized to the two-cycle study protocol are given in Table 1. Age range was from 24 to 58 years, and BMI from 30.4 to 92.5 kg/m^2^. All participants were Hispanic. There were 3 men and 13 women. Six participants were taking only oral antidiabetic agents, while ten were also taking insulin.

**Table 1:** Demographics and baseline characteristics for all enrolled participants.

ID	Age (years)	Gender (M;F)	Duration diabetes (years)	Height (m)	Weight (kg)	BMI (kg/m^2^)	Initial A1C (%)	Initial insulin (units/day)	Initial glimepiride (mg/d)	Initial metformin (g/d)	Initial pioglitazone (mg/d)
1	31	F	10	1.62	129.8	49.5	7.4	150	0	2	0
2	45	F	4	1.56	114.2	46.9	7.2	0	0	2	0
3	58	F	28	1.56	122.0	50.5	8.5	20	4	2	15
4	49	F	27	1.44	95.8	46.2	8.3	50	0	2	0
5	37	F	17	1.56	124.8	51.6	9.1	210	0	0.5	0
6	41	M	5	1.73	275.3	92.5	7.3	0	4	2	45
7	24	F	13	1.64	85.9	31.9	8.6	0	2	2	0
8	49	F	3	1.55	112.0	46.6	8.5	18	8	2	0
9	50	M	7	1.67	162.1	58.1	9.2	140	0	2	0
10	30	F	13	1.70	87.8	30.4	11.3	85	0	2	0
11	42	F	4	1.59	103.5	40.9	7	0	0	2	0
12	50	F	5	1.59	116.1	45.9	6.2	0	8	2	15
13	45	F	14	1.56	74.2	30.5	6.8	120	0	2	0
14	30	F	2	1.59	88.8	35.1	11	70	0	2	0
15	45	M	9	1.69	134.9	47.2	8.4	28	8	2	0
16	37	F	4	1.66	113.0	41.0	7.4	0	0	0.5	0
Avg	41.4	3 M:13 F	10.3	1.6	121.3	46.6	8.3	55.7	2.1	1.8	4.7
SD	9.3		8.1	0.1	46.5	14.7	1.4	66.9	3.2	0.5	11.9

### Safety

There were no significant clinical or device-related adverse events reported throughout the study, including the 334 days of tube use. Minor discomfort related to the oral anchor was the most frequently reported concern. Participants were inconsistent with providing glucose readings, and flash glucose monitoring was used for only a few cycles with incomplete data collection. No participant reported or was noted to have any episode of symptomatic hypoglycemia or hyperglycemia, and no significant adverse events were noted related to glucose control.

### Device

Device-related adverse events for each participant including all harms and unintended effects are given in Table 2. Two tubes were removed the day of initial placement and one after 5 days, due to poor tolerance. Eight of the nine participants who had the tube placed for ≥1 day tolerated it well, and six elected to participate in multiple 14-day cycles of use. Several participants reported episodes of difficulty administering treatment. They generally overcame this problem themselves by partially withdrawing the tube and reinserting it as advised, and they became more confident in adjusting the tube position in second and later cycles. On several occasions, the participants removed the tube themselves without incident and with rapid relief of any associated problems.

**Table 2:** Details of treatment cycles and device-related events for all enrolled participants.

MS ID	Order in study	Days with tube (total #)	Cycles done (total #)	Anchor issues (Y/N)	Tube issues (Y/N)	Wished to continue (Y/N)	Reason for stopping	Device-related issues
1	3	0	0	N	NA	N	Too busy	Minor buccal mucosal irritation.
2	9	0	0	Y	NA	N	Unable to place anchor	Unable to place anchor comfortably on low-profile molar.
3	13	0	0	Y	NA	Y	Unable to tolerate anchor	Shallow buccal mucosal ulcer due to mucosa lying on sharp edge of channel on single isolated molar.
4	15	0	0	NA	NA	Y	End of the study	None
5	11	0	0	N	Y	N	Anxiety after tube placement and test MM	Discomfort at anchor site in mouth on tube placement.
6	2	0.5	0	N	Y	N	Gagging on reclining at home first evening	Reported tube riding up into throat possibly due to high intra-abdominal pressure with BMI of 92.5 kg/m^2^.
7	16	5	0.2	Y	Y	N	Multiple minor problems, including busy schedule and anxiety after tube placement and test MM.	Discomfort at anchor site in mouth on tube placement, sore throat for 5 days, unable to administer feeds, likely due to kinked tube.
8	4	7	0.25	Y	N	Y	Unable to tolerate anchor after 7 days.	Shallow buccal mucosal ulcer due to mucosa lying on sharp edge of channel on single isolated molar.
9	6	14	1	N	N	N	Intercurrent unrelated clinical tests	None
10	10	14	1	N	N	N	Non-adherent to intervention	None
11	8	28	2	N	N	Y	Too busy	None
12	12	42	3	N	N	Y	End of the study	Tube removed by participant on one occasion after gagging while brushing teeth. Replaced next day without incident.
13	14	42	3	N	N	Y	End of the study	None
14	7	56	4	N	N	Y	Too busy	Tube removed by study team 1 day early on 1 occasion as precaution. Participant had lower back pain likely due to unrelated urinary tract infection.
15	1	56	4	N	N	Y	End of the study	Participant repeatedly bit on tube while eating. On one occasion, this caused a small perforation. Thus, tube replaced prophylactically each week.
16	5	70	5	Y	Y	Y	Too busy	Channel developed calculus between two cycles requiring reaming out by dentist. Participant felt nauseous twice after strong cooking smells, leading to tube recoil and removal by the participant.
Avg (SD) for all enrollees		20.9 (24.4)	1.5 (1.8)	Y: 5	Y:4			
				N:10	N:8	Y:9		
Avg (SD) for 10 enrollees with tube > 1 day		33.4 (23.1)	2.4 (1.7)	NA:1	NA:4	N:7		
Total		334	23.8					

### Primary and secondary outcomes in weight change

**Fig. 2:**
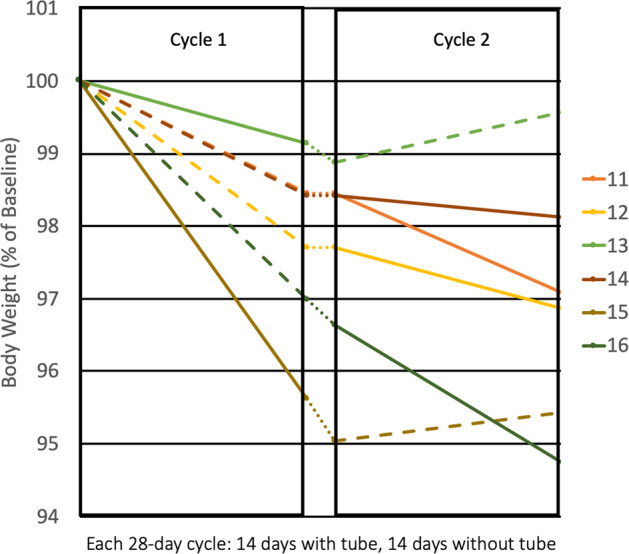
Weight loss for six participants undergoing at least two cycles. Lines: Solid, mixed meal; dashed, electrolyte solution; dotted, between cycles. Each 28-day cycle included: (1) 14 days with tube in situ and tube feeds; (2) 14 days follow-up without tube in situ.Between cycle interval: (1) Not to scale for time; (2) range: 0 day to 4 months.

For each primary (14-day weight loss) and secondary outcome (28-day weight loss, post 14-day weight loss), results for each group are reported as mean (95% confidence interval [CI]) weight loss (in kg) in Table 3 (Table 3, Fig. 2). All analyses were by original assigned treatment. In the two-cycle per protocol analysis, participants lost an average of −2.07 kg (95% CI: −3.26, −0.89) on MM cycles (within-MM cycle, *p* = 0.008) while ES cycles showed a lower average weight loss of −0.69 kg (−1.87, 0.49) (within-ES cycle, *p* = 0.18; between MM/ES cycle, *p* = 0.082). Similar results were noted for weight change measured from beginning to end of the 4-week cycles and there was no weight change in the post 14-day (day 14–28) cycle period (Table 3).

**Table 3:** Change in weight over 4-week cycles in six participants: electrolyte solution (ES) vs mixed meal (MM).

	Electrolyte solution (ES) mean (95% CI)^a^	Mixed-meal (MM) mean (95% CI)^a^	Difference (MM minus ES) mean (95% CI)	*p*-value between interventions
*Per protocol analysis*^b^				
2-week change from cycle baseline	−0.69 (−1.87, 0.49)	−2.07 (−3.26, −0.89)	−1.39 (−3.06, 0.28)	0.082
* p*-value^c^	0.18	0.008		
4-week change from cycle baseline	−0.64 (−2.01, 0.74)	−2.40 (−3.78, −1.02)	−1.76 (−3.71, 0.18)	0.066
* p*-value^c^	0.27	0.008		
2- to 4-week change	0.05 (−1.66, 1.76)	−0.32 (−2.03, 1.38)	−0.37 (−2.79, 2.04)	0.69
* p*-value^c^	0.94	0.63		
*Exploratory analysis*^b^				
2-week change from cycle baseline	−0.19 (−1.77, 1.39)	−1.58 (−2.52, −0.64)	−1.39 (−3.02, 0.25)	0.085
* p*-value^c^	0.78	0.005		
4-week change from cycle baseline	0.45 (−1.11, 2.01)	−1.31 (−2.29, −0.33)	−1.76 (−3.33, −0.19)	0.033
* p*-value^c^	0.51	0.017		
2- to 4-week change	0.63 (−1.91, 3.17)	0.25 (−1.34, 1.84)	−0.37 (−2.93, 2.18)	0.73
* p*-value^c^	0.57	0.71		

^a^Analyzed by general linear model, main effects for crossover sequence, cycle number (period), intervention (mixed meal, electrolyte), with random effect for subjects nested within sequence. Numbers in table are least-square means (95% confidence interval) by treatment, adjusted for sequence and cycle.

^b^Per protocol analysis data set includes 12 cycles (6 mixed meal, 6 electrolyte) in six subjects. Exploratory analysis data set includes 18 cycles (12 mixed meal, 6 electrolyte) in six subjects.

^c^Within intervention *p*-value, testing weight change differs from zero.

### Other exploratory clinical outcomes

Six additional optional MM cycles were completed in four of the participants (Table 3). A comparison of weight change between the 6 ES and 12 MM cycles was conducted in an exploratory analysis. As in the pre-specified analyses, data again showed that MM was associated with statistically significant weight loss at both 2 and 4 weeks, while ES was not. In this analysis, the difference in weight loss was significant between ES and MM at 4 weeks (*p* = 0.033), but not at 2 weeks.

More detailed findings have been reported in abstract case reports in two of these participants who each completed a total of four cycles^25,26^.

## Discussion

In this study, we demonstrated the feasibility of administering repeated enteral boluses of nutrients in a minimally invasive, secure, and unobtrusive manner via a novel modified orojejunal feeding tube. We also aimed to evaluate for the first time, the effect of repeated jejunal boluses on weight in ambulatory adults with obesity and type 2 diabetes. We observed significant weight loss from beginning to end of MM administration, but no significant difference in weight change with ingestion of higher calorie MM and lower calories ES treatments, with no significant differences between interventions.

The technique used has low feasibility for widespread adoption. Only a small proportion of participants screened for participation completed this pilot study, as also reported by other researchers when conducting pilot trials of novel weight-loss devices^27^. However, the lessons learned from this pilot study can be used to significantly improve recruitment and retention rates going forward. For example, self-referral in response to flyers and discussing participation with individuals already actively seeking weight loss was more successful than approaching participants based on medical criteria. The absence of any adverse events in almost a year of device use suggests that BMI, age, and medical eligibility criteria may be broadened, with the frequency of study visits decreased. Furthermore, populations obtaining routine dental healthcare and younger individuals without diabetes-related periodontal disease or tooth loss will have a higher chance of meeting dental eligibility requirements^28^. Within the study, the proactive use of orthodontic wax was found to reduce mucosal irritation by the dental anchor and increased successful participation in the study. Apprehension in participants regarding tube use can be reduced by extensive pre-procedure discussion, allowing participants to become used to the anchor prior to tube placement, and by the use of a low-calorie initial test feed to minimize any dumping symptoms. Although we did not use any analgesia during tube placement, this may also allow for easier tube placement in some participants.

We included individuals with obesity and type 2 diabetes, rather than obesity only, for two main reasons. First, given the relatively short duration of the treatments (2-week cycles), we considered that effects might be seen on glucose levels but not on weight. This hypothesis was based on studies suggesting weight-independent improvement in glucose early after intervention^12^. Therefore, demonstration of this could provide support for further work on this important topic. Second, prior work had included adults with obesity and type 2 diabetes, hence we elected to continue with the same study population. Future studies should evaluate the effect of bolus jejunal feeding in individuals with obesity only.

The weight loss observed in this study occurred without any request to participants that they restrict their nutrient intake. Net weight loss occurred following administration of the ES selected as a “control” treatment. Jejunal distension may have led to some satiation with this treatment, consistent with reports of decreased ghrelin production with duodenal infusion of hyperosmolar saline^29^. Continued weight loss in the 2 weeks after removal of the tube was noted in some participants, which may reflect persistent changes to the duodenal or jejunal mucosa, including in enteroendocrine cells and hormone production^30^.

There were several major limitations to this study. The position of the tube tip was not confirmed after initial placement due to removal of the dual-access port which precluded reinsertion of the electromagnetic stylet. For proof-of-concept, the location of the tube tip should be confirmed after placement. It is certainly possible that some tubes could have coiled in the stomach, although expert opinion was that in ambulatory adults with no significant GI impairment, the tube would likely progress aborally due to usual peristaltic forces. In addition, adherence to nutrient administration was not formally evaluated. Thus, it is not known what amount of the prescribed treatment was actually delivered to the jejunum. Furthermore, medications were adjusted at the clinical discretion of the PI, which may have introduced bias to the results. Numerous desirable metrics were not collected. Future studies should evaluate food intake, gut hormones, appetite and gut sensations, and gut transit to understand the mechanisms of any intervention effects and improve on the technique. Safe and accurate monitoring of glucose levels will now be greatly facilitated by the wide availability of continuous glucose monitoring.

The lack of blinding in this study was sub-optimal. This step was taken to safely provide nutrient of desired composition to the participant. Several steps to mitigate the lack of blinding were taken, including no discussion with the participant regarding possible differing effects of the nutrients, removal of identifying labels, not referring to the participant as being in a “control” or “intervention” cycle, and assessment of the main outcome measure by the research assistant. Furthermore, the investigators were not blinded to the type of infusion, though steps were taken to minimize the impact of this on weight measurements. In future studies, double blinding should be used to minimize risk of bias.

Future studies should aim to assess the position of the tube tip during use and to standardize the amount, timing, and rate of the selected nutrient administration. Once the response to a consistently administered stimulus at a consistent site has been determined, then the stimulus type, amount, and site of administration can be iteratively altered to optimize therapeutic efficacy and inform the development of other nutrient-based weight-loss strategies, including caloric restriction, and functional foods such as enteric-coated nutrients for release in the distal intestine^31–36^.

## Conclusion

We demonstrated that repeated bolus nutrient administration for up to 2 weeks at a time via a novel, intraorally anchored enteral feeding tube is feasible. The prototype device requires further development to allow rigorous evaluation of the clinical value of bolus jejunal feeding for weight management, obesity-related comorbidities, and the development of other nutrient-based therapies.

## Supplementary Information

Supplementary Information
